# Protective effect of gut microbiota restored by fecal microbiota transplantation in a sepsis model in juvenile mice

**DOI:** 10.3389/fimmu.2024.1451356

**Published:** 2024-10-22

**Authors:** Young Joo Han, SungSu Kim, Haksup Shin, Hyun Woo Kim, June Dong Park

**Affiliations:** ^1^ Department of Pediatrics, Yonsei University College of Medicine, Seoul, Republic of Korea; ^2^ Laboratory Animal Experiment Center, Bionsystems, Uiwang-si, Gyeonggi-do, Republic of Korea; ^3^ Wide River Institute of Immunology, Seoul National University, Hongcheon-Gun, Gangwon-do, Republic of Korea; ^4^ Bio Convergence Team, Gangwon Techno Park Technology Innovation Support Center, Chuncheon-si, Gangwon-do, Republic of Korea; ^5^ Department of Pediatrics, Seoul National University College of Medicine, Seoul, Republic of Korea

**Keywords:** fecal microbiota transplantation, sepsis, microbiota, antibiotics, mouse model, pediatric

## Abstract

**Introduction:**

Restoring a balanced, healthy gut microbiota through fecal microbiota transplantation (FMT) has the potential to be a treatment option for sepsis, despite the current lack of evidence. This study aimed to investigate the effect of FMT on sepsis in relation to the gut microbiota through a sepsis model in juvenile mice.

**Methods:**

Three-week-old male mice were divided into three groups: the antibiotic treatment (ABX), ABX-FMT, and control groups. The ABX and ABX-FMT groups received antibiotics for seven days. FMT was performed through oral gavage in the ABX-FMT group over the subsequent seven days. On day 14, all mice underwent cecal ligation and puncture (CLP) to induce abdominal sepsis. Blood cytokine levels and the composition of fecal microbiota were analyzed, and survival was monitored for seven days post-CLP.

**Results:**

Initially, the fecal microbiota was predominantly composed of the phyla Bacteroidetes and Firmicutes. After antibiotic intake, an extreme predominance of the class Bacilli emerged. FMT successfully restored antibiotic-induced fecal dysbiosis. After CLP, the phylum Bacteroidetes became extremely dominant in the ABX-FMT and control groups. Alpha diversity of the microbiota decreased after antibiotic intake, was restored after FMT, and decreased again following CLP. In the ABX group, the concentrations of interleukin-1β (IL-1β), IL-2, IL-6, IL-10, granulocyte macrophage colony-stimulating factor, tumor necrosis factor-α, and C-X-C motif chemokine ligand 1 increased more rapidly and to a higher degree compared to other groups. The survival rate in the ABX group was significantly lower (20.0%) compared to other groups (85.7%).

**Conclusion:**

FMT-induced microbiota restoration demonstrated a protective effect against sepsis. This study uniquely validates the effectiveness of FMT in a juvenile mouse sepsis model, offering potential implications for clinical research in critically ill children.

## 1 Introduction

A healthy gut microbiota is in symbiosis with the host, inhibits the colonization of pathological microorganisms, and contributes to immune regulation and homeostasis (1). The gut microbiota undergoes changes in various diseases, not limited to intestinal conditions. An unfavorable imbalance in the composition and diversity of gut microbiota is believed to play a role in severe diseases, including sepsis (2). Although the precise mechanisms by which the gut microbiota influences disease progression are not fully understood, it is clear that these imbalances can significantly impact health and diseases. If the integrity of the intestinal mucosa is compromised, translocation of gut microbiota can occur, further contributing to disease development (3).

Antibiotics, frequently used in patients with severe diseases, temporarily alter the host’s microbiota and affect immunity (3). Among pediatric intensive care unit (PICU) patients, 58 to 74% receive antibiotics (4–6). A study comparing the microbiota of PICU patients, where 89% received antibiotics, with that of healthy children and adults found reduced diversity and quantity of microbiota in PICU patients. Over 50% of their microbiota comprised dominant pathogens, and regional differences in microbiota were diminished (7).

Despite imposing a significant economic burden, sepsis maintains a high fatality rate, causing five million deaths annually worldwide (8, 9). Even in countries with advanced medical facilities, sepsis mortality remains high, particularly among children, with rates ranging from 21 to 40% (10). Host immune dysregulation in response to invasive infection is a known mechanism of sepsis (11). Sepsis guidelines emphasize prevention, early antibiotic administration, and maintaining effective perfusion (12–14). However, these guidelines have seen no groundbreaking changes in the past decade (15). Although non-clinical studies in immunology have proposed potential treatments for sepsis (16), recent large-scale clinical studies have found many of these approaches broadly ineffective (15). Therefore, further research is urgently needed to discover new therapeutic alternatives for sepsis.

The association of microbiota with diseases holds the potential to offer an alternative treatment option for sepsis. Particularly considering the high rate of antibiotic use among PICU patients, it is crucial to determine whether maintaining or restoring a healthy microbiota can contribute to sepsis treatment.

Although enteral nutrition and supplementation with prebiotics or probiotics have been suggested as treatments for maintaining or restoring a healthy gut microbiota for a substantial time (17, 18), fecal microbiota transplantation (FMT) appears to offer a more direct acquisition of composition and diversity resembling a healthy gut microbiota (19). In a study inoculating *Streptococcus pneumoniae* into mouse nasal cavities, the post-antibiotic FMT group, exhibited normalized pulmonary bacterial counts, tumor necrosis factor-α (TNF-α), and interleukin-10 (IL-10) levels (20). However, aside from studies in patients with *Clostridium difficile* colitis and a limited number of case reports, no large-scale clinical studies have been conducted to date on FMT in critically ill patients (21–24).

Serious complications of FMT performed for *Clostridium difficile* colitis are infrequent (25). However, there is a risk of translocation infection from donor microorganisms, especially in immunocompromised patients or when microorganisms are aspirated during FMT via the upper gastrointestinal tract. When administered via colonoscopy, potential complications of the procedure must also be considered. Therefore, more experimental evidence is essential for designing clinical studies. Specifically, for FMT research in pediatric sepsis, prior investigations into specific indications, timing, duration, dosage, and potential serious complications are necessary.

This study aimed to investigate whether therapeutically acquired gut microbiota through FMT, following antibiotic disruption of existing microbiota, exerts a protective effect in a sepsis model in juvenile mice. The findings will be compared with existing non-clinical and clinical research and will inform the design of clinical studies targeting children with serious diseases, including sepsis.

## 2 Methods

**Figure 1:**
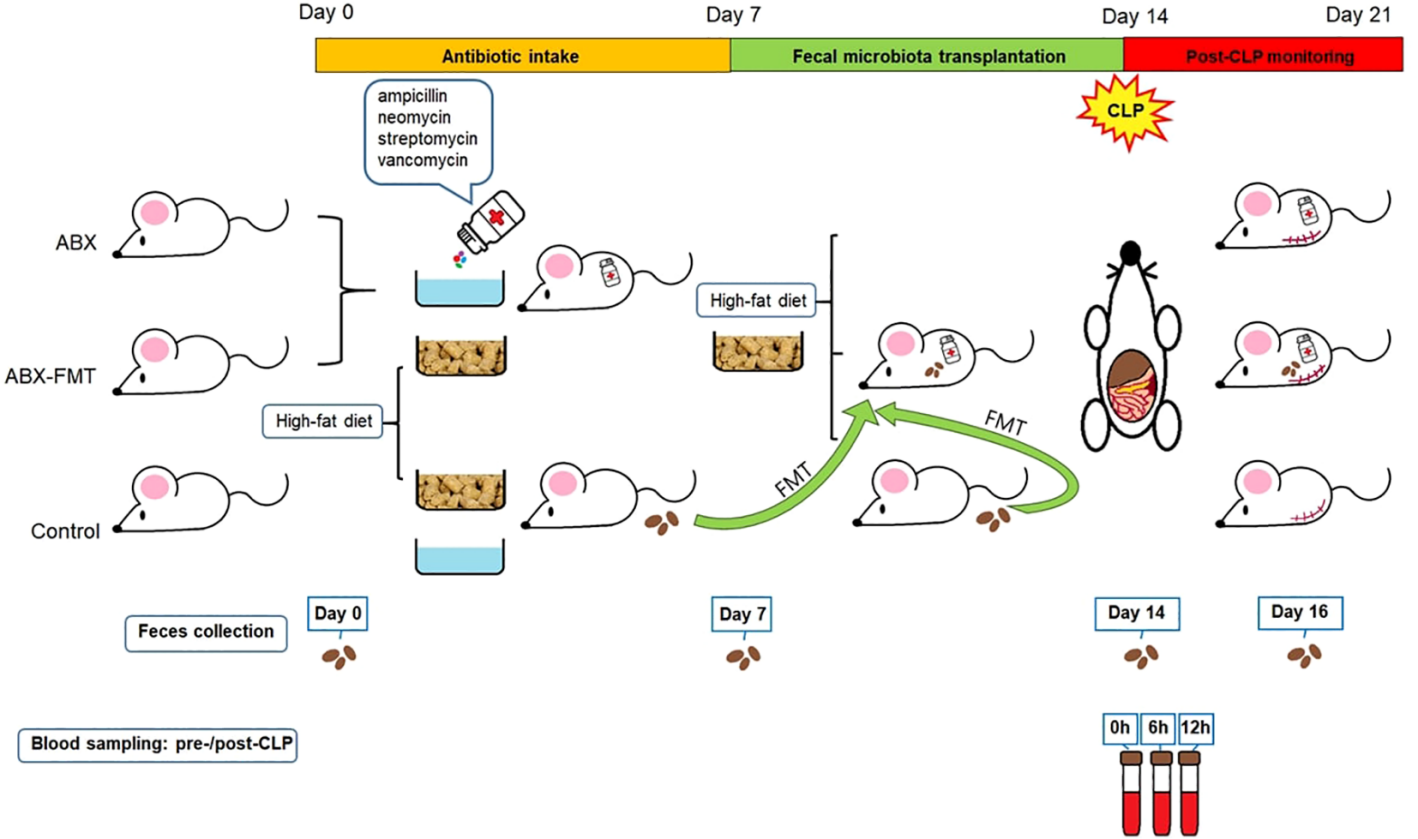
Study design. ABX, antibiotic treatment; CLP, cecal ligation and puncture; FMT, fecal microbiota transplantation.

The experimental process for this study was conducted in collaboration with Wide River Institute of Immunology, Seoul National University, Korea. Pathological staining and microscopic interpretation of mouse organ tissues were carried out in cooperation with the Korea Non-Clinical Technology Solution Center, Korea. A schematic summary of the study design is presented in **Figure 1**. Detailed methods are provided in the online supplement.

### 2.1 Experimental animals

Three-week-old male C57BL/6NCrlOri mice, specific pathogen-free, obtained from Charles River Laboratories in Wilmington, MA, USA, were used. After a three-day acclimatization period, animal studies were conducted in accordance with the Institutional Animal Care and Use Committee of Seoul National University College of Medicine (SNU-191010-5-1 and SNU-210514-1).

To prevent weight stagnation or loss, which could directly impact survival in growing mice, a high-fat diet (TD.06414, Envigo, Indianapolis, IN, USA: 60% fat, 20% protein, 20% carbohydrate; 5.1 kcal/g) was administered from the experiment’s initiation until the cecal ligation and puncture (CLP) procedure. Subsequently, all mice were switched to a standard diet.

### 2.2 Antibiotic treatment

Mice in the ABX or ABX-FMT group were given a sterile-water drink containing four antibiotics (ampicillin 1 g/L, neomycin 1 g/L, streptomycin 1 g/L, and vancomycin 0.5 g/L; all from Sigma Aldrich, St. Louis, MO, USA) for seven days starting from the initiation of the experiment (day 0).

### 2.3 FMT

Mice from the control group served as transplant donors. Fecal pellets, freshly collected under sterile conditions during defecation, were resuspended in phosphate-buffered saline (PBS) at a ratio of one fecal pellet per 1 mL of PBS, and then vortexed. FMT was administered once a day for seven consecutive days, starting on the 8th day of the experiment (day 7), coinciding with the conclusion of antibiotic intake. Recipient mice in the ABX-FMT group fasted for 30 minutes before receiving fecal supernatant from at least three individual donor mice via oral gavage at a dose of 500 mg/kg based on body weight. In contrast, ABX or control group mice, also fasting for 30 minutes, received Dulbecco’s PBS using the same oral gavage procedure (500 mg/kg).

### 2.4 CLP procedure

After the completion of FMT, on the 15th day of the experiment (day 14), CLP was performed on all mice according to general guidelines (26, 27). After the surgery, body temperature was maintained using an infrared lamp for up to six hours post-CLP. The death of each subject was confirmed at three, four, six, 18, and 24 hours, and subsequently every 24 hours until seven days after CLP (day 21). Deaths occurring 24 hours post-CLP were attributed to CLP-induced sepsis.

### 2.5 Blood collection and analysis of inflammatory biomarkers

Blood sampling was conducted three times: immediately before CLP and at six and 12 hours post-CLP. Cytokines, including IL-1β, IL-2, IL-4, IL-6, IL-10, interferon-γ (IFN-γ), TNF-α, granulocyte macrophage colony-stimulating factor (GM-CSF), and C-X-C motif chemokine ligand 1 (CXCL1), were quantified using a multiplex immunoassay conducted on the Bio-Plex 200 system (Bio-Rad, Hercules, CA, USA) (28).

### 2.6 Stool collection and bacterial DNA extraction, PCR amplification, and sequencing

Stool from all mice were collected on day 0, day 7, day 14, and day 16. DNA extractions were conducted using the QIAamp DNA Stool Mini Kit (Qiagen, Germantown, MD, USA) following the manufacturer’s instructions (29, 30). The yield of extracted DNA was determined using a NanoDrop 2000 spectrophotomether and a Qubit 3.0 fluorometer, both supplied by Thermo Fisher Scientific, Waltham, MA, USA (29).

The extracted DNA was used as a template for amplicon PCR targeting the V3 and V4 regions of the bacterial 16S rRNA gene. The 16S metagenomic sequencing library was prepared following the Illumina 16S Metagenomic Sequencing Library Preparation protocol (Illumina, San Diego, CA, USA) (29). Each PCR was duplicated using primer pairs with Illumina overhang sequences and inner tags: forward 5’-CGTCGGCAGCGTCAGATGTGTATAAGAGACAGCCTACGGGNGGCWGCAG-3’ and reverse 5’-GTCTCGTGGGCTCGGAGATGTGTATAAGAGACAGGACTACHVGGGTATCTAATCC-3. After the 2 × 250 bp Illumina MiSeq paired-end sequencing run, base-calling was performed, and reads with the same barcode were collected and assigned to their respective samples on the instrument, generating Illumina FASTQ files (29).

### 2.7 Extraction of the small intestine and histological examination

After sacrificing the surviving mice seven days post-CLP, the small intestines were surgically separated, washed with PBS, and fixed in 10% neutral-buffered formalin (Sigma, HT501128). Automated tissue processing and paraffin embedding were then performed, followed by sectioning of the paraffin blocks into 3 µm thick slices using a microtome. The sections were mounted on glass slides and stained with hematoxylin and eosin (H&E). The stained small intestines were examined under a light microscope (BX53, Olympus, Japan). Lesions observed in the small intestines and mesentery were recorded and semi-quantitatively evaluated based on their severity, with categorizations into four grades: minimal, mild, moderate, and severe.

### 2.8 Statistical analyses

Data are presented as mean ± standard deviation. Statistical analysis employed GraphPad Prism version 9.5.1 (GraphPad Software, Boston, MA, USA). Alpha diversity (Shannon and Simpson’s indices) was assessed using Excel (Microsoft, Redmond, WA, USA). Beta diversity was analyzed via non-metric multidimensional scaling (NMDS) plots based on Bray-Curtis or Euclidean distances for dissimilarity using XLSTAT version 2023.2.1 (Lumivero, Denver, CO, USA). Kaplan-Meier survival curves were compared using the logrank (Mantel-Cox) test.

A two-tailed Welch’s t-test was used to compare two means, while a Brown-Forsythe/Welch analysis of variance with Dunnett’s T3 multiple comparisons test was employed to analyze differences among three or more means. To clearly contrast differences between specific groups, Welch’s t-test results are emphasized in the Results section. This approach was applied to the analysis of the relative abundance of bacteria, the Shannon index, and cytokine levels. For significant comparisons between two means, effect sizes (Cohen’s d) and their 95% confidence intervals were calculated using Excel. The Mann-Whitney U test was used to compare the categorization of pathological inflammation severity between groups. Statistical significance was set at p < 0.05.

## 3 Results

### 3.1 Initial fecal microbiota

**Figure 2:**
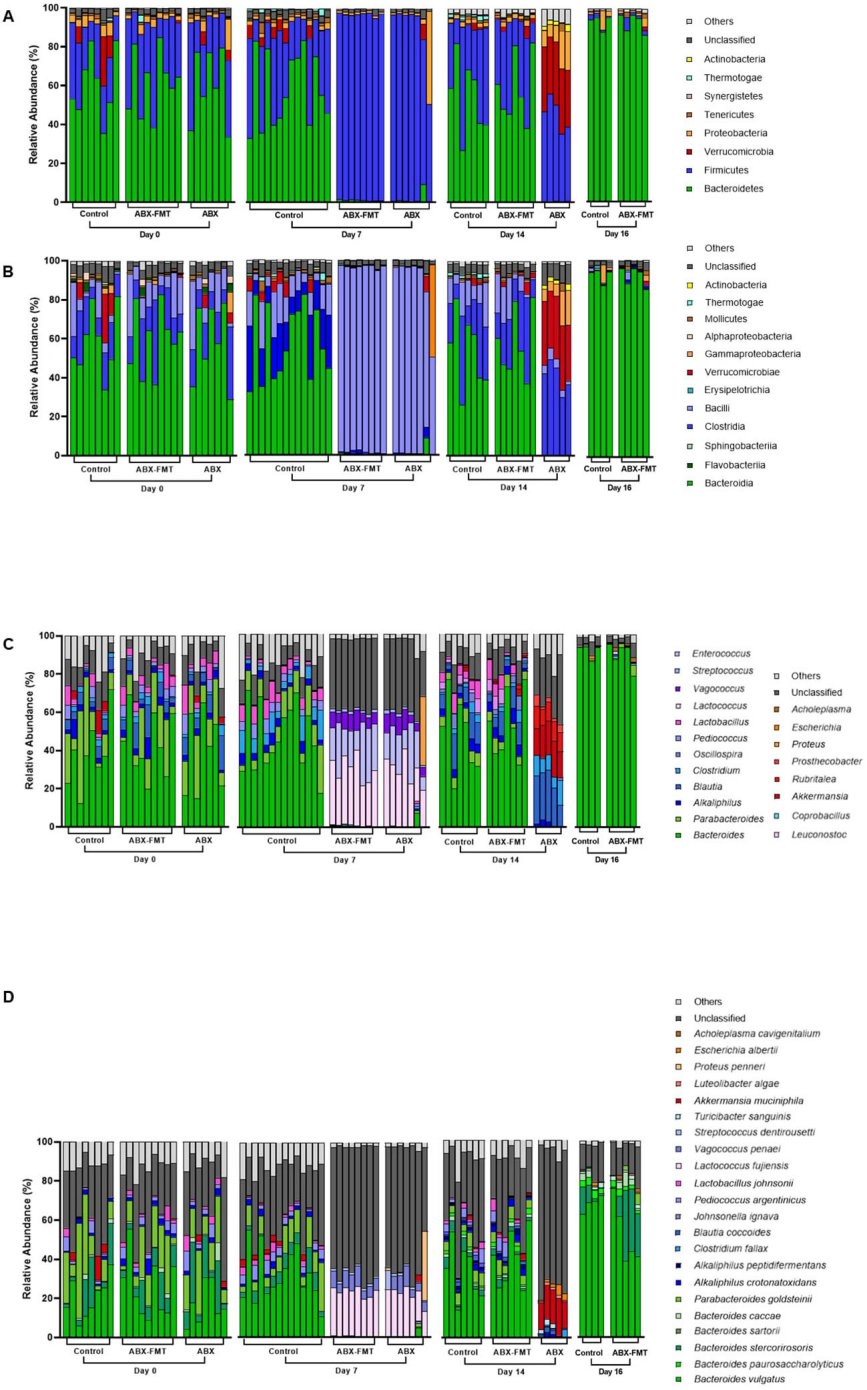
Composition of fecal microbiota plotted by group and experimental day. **(A)** Composition of fecal microbiota at the phylum level. **(B)** Composition of fecal microbiota at the class level. **(C)** Composition of fecal microbiota at the genus level. **(D)** Composition of fecal microbiota at the species level. ABX, antibiotic treatment; FMT, fecal microbiota transplantation.

The initial fecal microbiota (on day 0) in all subjects exhibited dominant phyla (> 1%), including Bacteroidetes (60.7%), Firmicutes (28.8%), Proteobacteria (3.7%), and Verrucomicrobia (2.5%). The phylum-, class-, genus-, and species-level composition of stool microbiota on day 0 showed no differences between each group (**Figure 2**).

### 3.2 Fecal microbiota after seven days of antibiotic intake

Based on measured water intake mixed with antibiotics, mice in the ABX group received 98.6 mg/kg/day of ampicillin, neomycin, and streptomycin, and 49.3 mg/kg/day of vancomycin, while the ABX-FMT group received 105.8 mg/kg/day of ampicillin, neomycin, and streptomycin, and 52.9 mg/kg/day of vancomycin, both for seven days.

**Figure 3:**
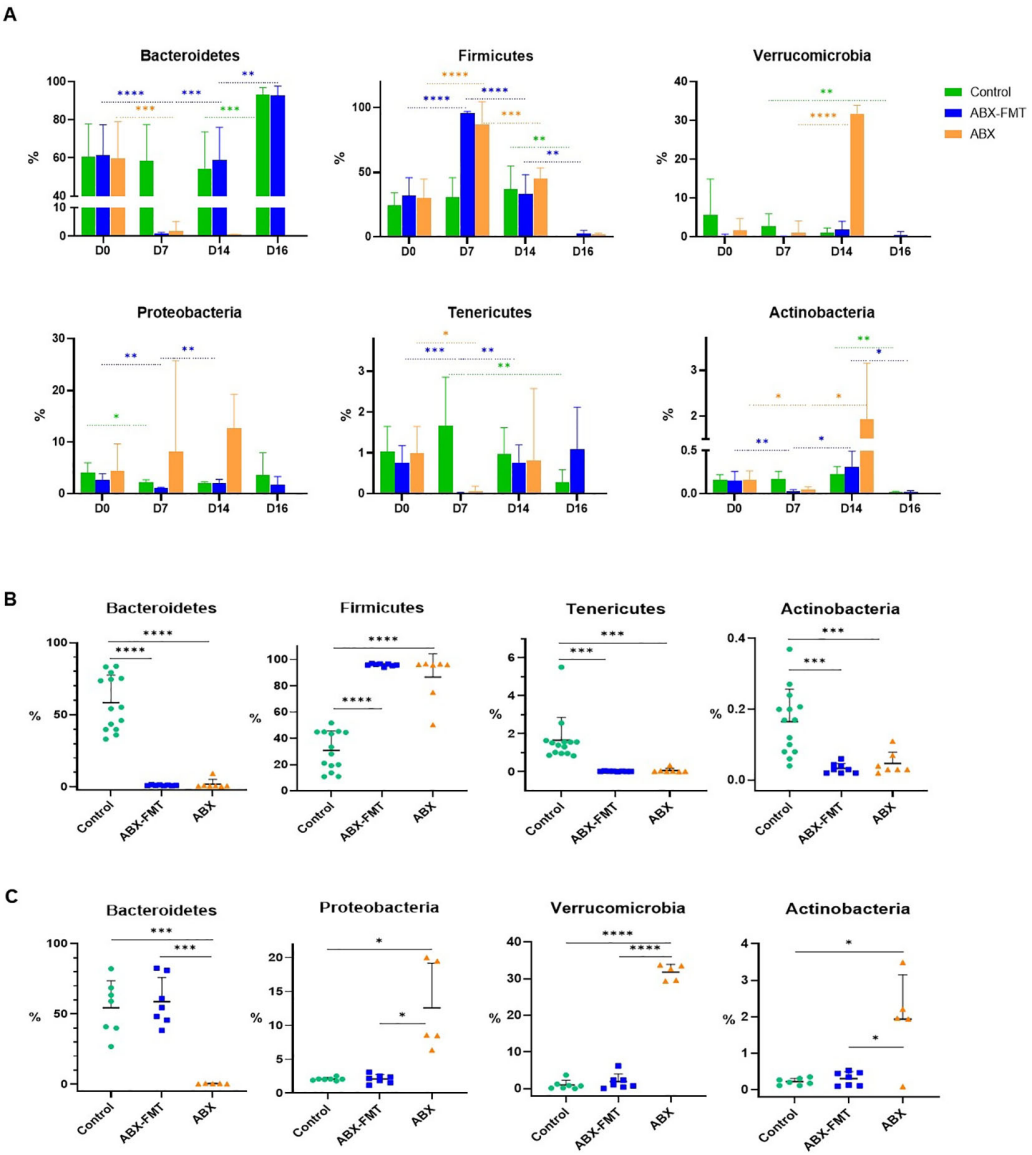
Relative abundance of each dominant phylum in the fecal microbiota of each group. The bar graph displays the mean values for each group, with error bars representing the mean plus one standard deviation. The y-axis represents the relative abundance of each phylum. **(A)** Changes in the abundance of each dominant phylum of fecal microbiota over time in each group. **(B)** Comparison of the relative abundance of each dominant phylum constituting the fecal microbiota on day 7 between each group. **(C)** Comparison of the relative abundance of each dominant phylum constituting the fecal microbiota on day 14 between each group. ABX: antibiotic treatment, FMT: fecal microbiota transplantation. Asterisks indicate statistical significance: *p < 0.05, **p < 0.01, ***p < 0.001, ****p < 0.0001 with two-tailed Welch’s t-test.

Fecal microbiota on day 7 was analyzed to assess the impact of antibiotic intake (**Figure 2**). Notably, significant differences in fecal microbiota between day 0 and day 7 were observed in the ABX group. On day 7, the ABX group exhibited higher relative abundance in various taxa, including *p:Firmicutes, c:Bacilli*, *g:Enterococcus*, *Lactococcus fujiensis*, and *Vagococcus penaei* compared to day 0. Conversely, there was a lower relative abundance of taxa, including *p:Bacteroidetes, p:Tenericutes, p:Actinobacteria*, *c:Clostridia*, *Bacteroides vulgatus*, *Bacteroides stercorirosoris*, *Parabacteroides goldsteinii*, *Pediococcus argentinicus*, and *Lactobacillus johnsonii* on day 7 compared to day 0 (**Figure 3A** and **Supplementary Table S1**). In the ABX-FMT group, changes in fecal microbiota between day 0 and day 7 mirrored those in the ABX group (**Figure 3A** and **Supplementary Table S2**). Conversely, the control group showed no significant changes in fecal microbiota, except for a lower relative abundance of *p:Proteobacteria* on day 7 compared to day 0 (**Figure 3A** and **Supplementary Table S3**).

Given the similarity in the day-0 microbiota of all groups and the day-7 microbiota of the control group, the distinction between the control group and the ABX or ABX-FMT group on day 7 mirrored the aforementioned changes observed in the ABX group before and after antibiotic administration (**Figure 3B** and **Supplementary Table S4**).

### 3.3 Fecal microbiota after seven days of FMT *vs*. no intervention following antibiotic intake

The ABX-FMT group, undergoing seven days of antibiotic intake followed by seven days of FMT, exhibited the most complex changes in fecal microbiota (**Figure 2**). On day 14, there was a notable increase in the relative abundance of various taxa, including *p:Bacteroidetes, p:Proteobacteria, p:Tenericutes*, *p:Actinobacteria*, *c:Clostridia*, *Bacteroides vulgatus*, *Bacteroides stercorirosoris*, *Parabacteroides goldsteinii*, and *Alkaliphilus crotonatoxidans* compared to day 7. Conversely, on day 14, the relative abundance of *p:Firmicutes*, *c:Bacilli*, *g:Enterococcus*, *Lactococcus fujiensis*, and *Vagococcus penaei* in the fecal microbiota of the ABX-FMT group was lower compared to day 7 (**Figure 3A** and **Supplementary Table S2**).

Despite no further interventions, the ABX group exhibited additional changes in fecal microbiota (**Figure 2**). On day 14, there was a notable increase in the relative abundance of *p:Verrucomicrobia*, *p:Actinobacteria*, *c:Clostridia*, *g:Rubritalea, Akkermansia muciniphila*, and *Escherichia albertii* in the fecal microbiota of the ABX group compared to day 7. Conversely, the relative abundance of *p:Firmicutes, c:Bacilli*, *g:Enterococcus*, *Lactococcus fujiensis*, and *Vagococcus penaei* in the fecal microbiota of the ABX group was lower on day 14 compared to day 7 (**Figure 3A** and **Supplementary Table S1**).

On day 14, the fecal microbiota of the FMT-ABX group closely resembled that of the control group (FMT donor). In contrast, a noticeable difference in fecal microbiota between the ABX group and the ABX-FMT group became apparent. Specifically, the ABX group exhibited lower relative abundances of various taxa, including *p:Bacteroidetes*, *g:Alkaliphilus, g:Lactobacillus, Bacteroides vulgatus, Bacteroides stercorirosoris*, and *Parabacteroides goldsteinii* compared to both the ABX-FMT and control groups. Conversely, the relative abundance of *p:Verrucomicrobia, p:Proteobacteria p:Actinobacteria, c:Clostridia, g:Rubritalea, g:Prosthecobacter, Akkermansia muciniphila*, and *Escherichia albertii* was higher in the ABX group compared to both the ABX-FMT and control groups on day 14 (**Figure 3C** and **Supplementary Table S5**).

### 3.4 Fecal microbiota in CLP-induced sepsis after antibiotic intake, with or without subsequent FMTafter seven days of FMT *vs*. no intervention following antibiotic intake

On day 16, two days after CLP, changes in fecal microbiota, not evident in the preceding 14 days, manifested in the control group (**Figure 2**). Feces from the ABX group couldn’t be collected due to the high mortality rate and absent defecation of surviving subjects on day 16. On day 16, the control group exhibited an increase in the relative abundance of *p:Bacteroidetes* and *Bacteroides vulgatus* compared to day 14. Conversely, the relative abundance of *p:Firmicutes, p:Actinobacteria, Parabacteroides goldsteinii, Alkaliphilus crotonatoxidans*, *Pediococcus argentinicus*, and *Lactobacillus johnsonii* decreased on day 16 compared to day 14 (**Figure 3A** and **Supplementary Table S3**). During this period, changes in fecal microbiota in the ABX-FMT group mirrored those in the control group (**Figure 3A** and **Supplementary Table S2**).

### 3.5 Differences and changes in fecal microbiota diversity

**Figure 4:**
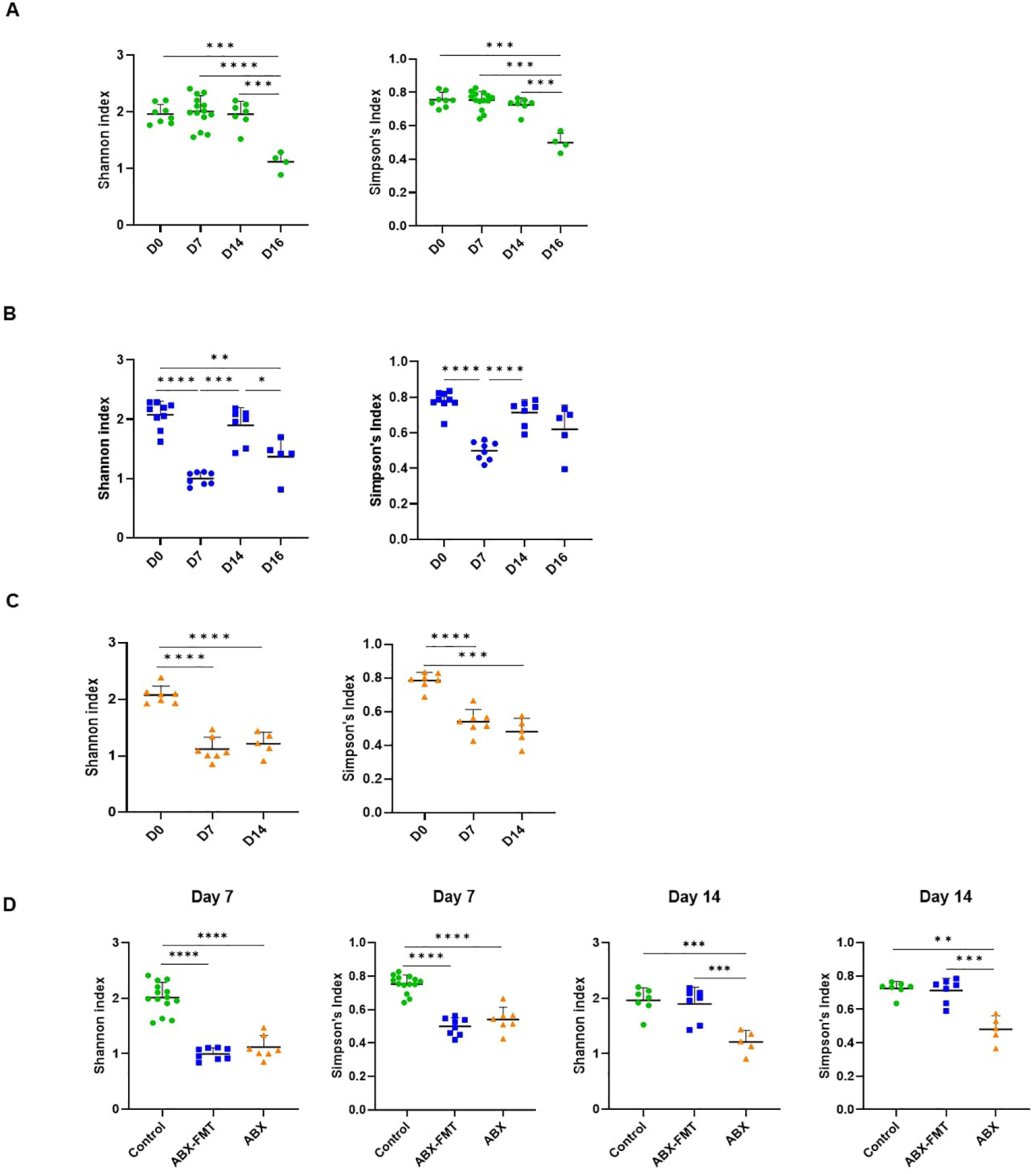
The Shannon and Simpson’s indices of fecal microbiota. Data are presented as individual values with long and short horizontal bars representing the mean plus one standard deviation. The y-axis represents either the Shannon or Simpson’s indices, depending on the specific panel. **(A)** Changes in the Shannon and Simpson’s indices for the control group over time. **(B)** Changes in the Shannon and Simpson’s indices for the ABX-FMT group over time. **(C)** Changes in the Shannon and Simpson’s indices for the ABX group over time. **(D)** Comparison of the Shannon or Simpson’s indices of fecal microbiota between each group on day 7 and day 14. ABX, antibiotic treatment; FMT, fecal microbiota transplantation. Asterisks indicate statistical significance: *p < 0.05, **p < 0.01, ***p < 0.001, ****p < 0.0001 with two-tailed Welch’s t-test.

In the control group, the Shannon and Simpson’s indices were lower only on day 16 compared to day 0, day 7, and day 14, indicating reduced alpha diversity after CLP (**Figure 4A**). In both ABX-FMT and ABX groups, these indices on day 7 decreased compared to day 0, suggesting reduced alpha diversity after antibiotic intake. In the ABX-FMT group, indices on day 14 increased compared to day 7, indicating recovery after FMT. However, after CLP, the Shannon index decreased again in the ABX-FMT group, similar to the control group. In the ABX group, indices on day 14 remained lower than those on day 0 (**Figures 4B, C**).

On day 0, there were no differences in Shannon and Simpson’s indices among the three groups. By day 7, both the ABX-FMT and ABX groups showed lower indices than the control group. On day 14, post-FMT, the ABX-FMT group showed no differences in Shannon and Simpson’s indices compared to the control group. However, the ABX group’s indices remained lower than those of the ABX-FMT and control groups (**Figure 4D**).

**Figure 5:**
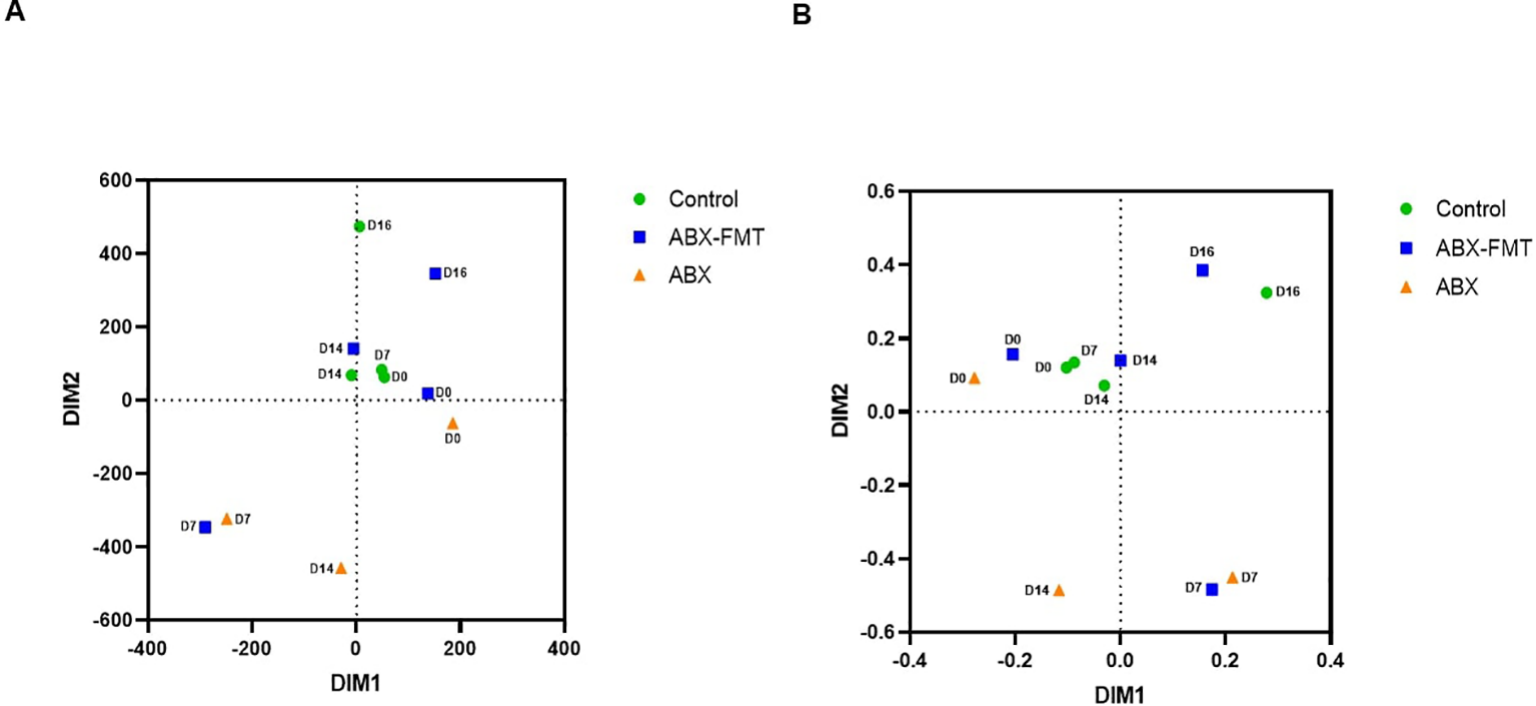
Beta diversity of the overall fecal microbiota. **(A)** Non-metric multidimensional scaling plots based on the Bray-Curtis distances for dissimilarity. **(B)** Non-metric multidimensional scaling plots based on the Euclidean distances for dissimilarity. ABX, antibiotic treatment, D0: day 0, D7: day 7, D14: day 14, D16: day 16, FMT, fecal microbiota transplantation.

NMDS plots, based on Bray-Curtis or Euclidean dissimilarity distances, depicted the contrast between the ABX group and the control group, as described above. The plots also illustrated similarities or dissimilarities between the ABX-FMT group and the ABX group or control group, depending on the time point (**Figure 5**).

### 3.6 Changes in serum cytokine concentrations after CLP

At pre-CLP, samples from two subjects in the ABX-FMT group were excluded due to errors. Serum concentrations of most cytokines in two out of seven subjects in the ABX-FMT group at pre-CLP exhibited significant variations from those in the other five subjects: IL-1β (mean for the two excluded subjects [pg/ml], 1393.8; mean for the other five subjects [pg/ml], 3.8), IL-6 (9486; 0.1), IL-10 (9669; 6.8), TNF-α (196, 0), GM-CSF (874, 0), and CXCL1 (24330; 9.1). Therefore, these samples were deemed unreliable due to potential contamination or deterioration and were excluded from the analyses of all pre-CLP cytokines in the ABX-FMT group.

**Figure 6:**
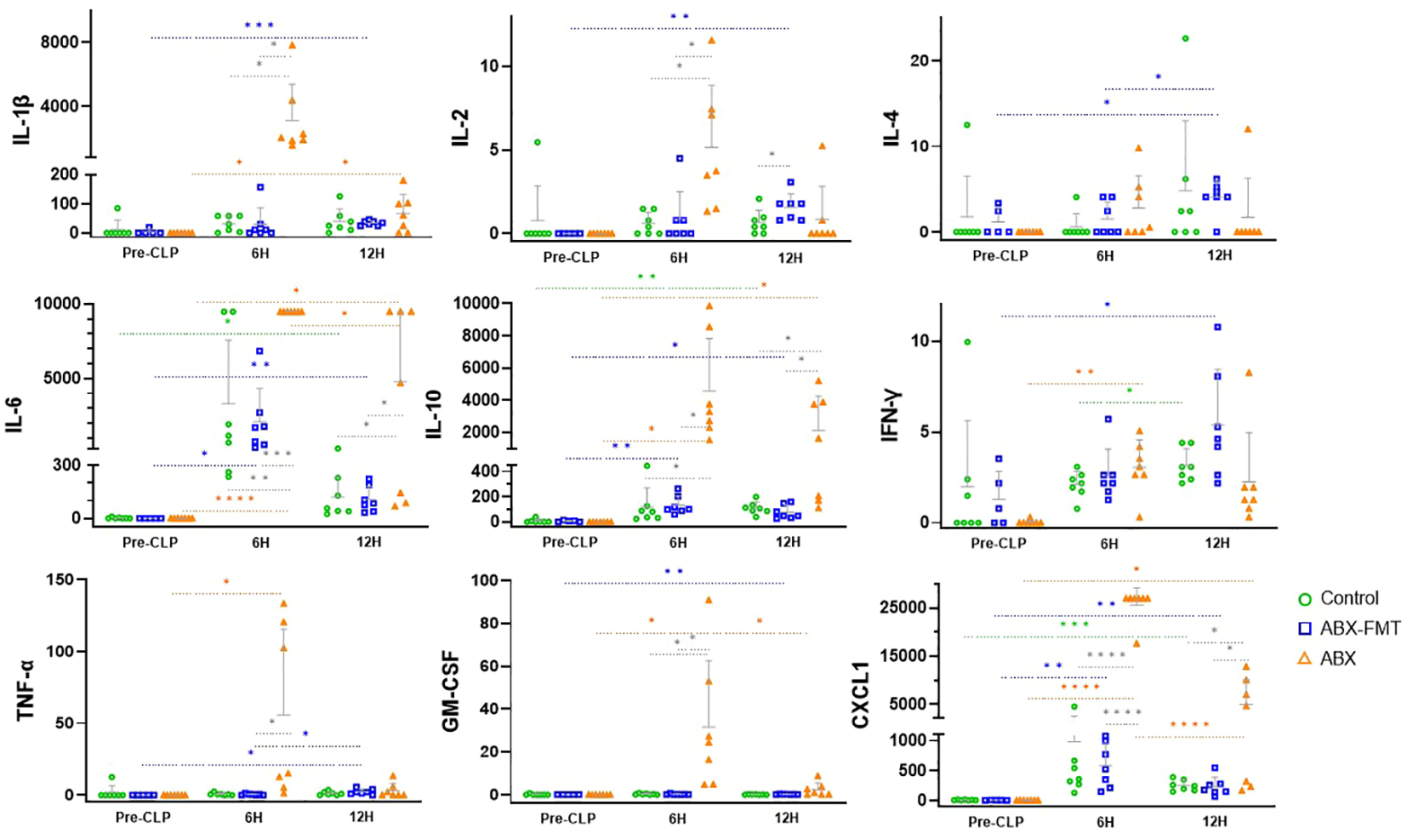
Changes in blood cytokine concentrations (pg/ml) in each group from immediately before CLP to 12 hours after. Grey horizontal bars indicate the mean values, and vertical lines represent the mean plus one standard deviation. Sample sizes: *n* = 7 at all time points in all groups, except for the ABX-FMT group at pre-CLP (*n* = 5, excluding two subjects due to sample errors). ABX, antibiotic treatment; CLP, cecal ligation and puncture; CXCL1, C-X-C motif chemokine ligand 1; FMT, fecal microbiota transplantation; GM-CSF, granulocyte macrophage colony-stimulating factor; IFN, interferon; IL, interleukin; TNF, tumor necrosis factor. Asterisks indicate statistical significance: *p < 0.05, **p < 0.01, ***p < 0.001, ****p < 0.0001 with two-tailed Welch’s t-test. Colored dotted lines represent comparisons across time within the same group, and solid black lines represent comparisons between groups at the same time.

The concentrations of IL-1β, IL-2, IL-4, IL-6, IL-10, IFN-γ, TNF-α, and GM-CSF measured immediately before CLP were found to be very low in the ABX group and higher in the control group, although this difference was not statistically significant. In the ABX group, the concentrations of IL-1β (pre-CLP *vs.* 6 h, *p* = 0.0111; 6 h *vs*. 12 h, *p* = 0.0122), IL-6 (*p* < 0.0001; *p* = 0.0379), IL-10 (*p* = 0.0101; NS), IFN-γ (*p* = 0.0017; NS), TNF-α (*p* = 0.0486; NS), GM-CSF (*p* = 0.035; *p* = 0.0456), and CXCL1 (*p* < 0.0001; *p* < 0.0001) increased six hours after CLP but decreased over the next six hours, except for IL-10, IFN-γ, and TNF-α. The ABX-FMT group exhibited a more gradual and relatively smaller increase in cytokines compared to the ABX group. Concentrations of IL-1β (*p* = 0.0002), IL-2 (*p* = 0.0022), IL-4 (*p* = 0.0192), IL-6 (*p* = 0.0083), IL-10 (*p* = 0.0126), IFN-γ (*p* = 0.0428), TNF-α (*p* = 0.02), GM-CSF (*p* = 0.0021), and CXCL1 (*p* = 0.0092) were higher 12 hours after CLP than before CLP. Among these, the concentrations of IL-6 (*p* = 0.0469), IL-10 (*p* = 0.0034), and CXCL1 (*p* = 0.0065) increased for six hours after CLP but did not decrease for the subsequent six hours. In the control group, 12 hours after CLP, IL-6 (*p* = 0.0323), IL-10 (*p* = 0.0011), and CXCL1 (*p* = 0.0004) exhibited increased concentrations compared to pre-CLP levels, and IFN-γ (*p* = 0.0337) showed a higher concentration than at six hours post-CLP (**Figure 6**).

Six hours after CLP, the concentrations of IL-1β (ABX *vs.* control, *p* = 0.0116; ABX *vs.* ABX-FMT, *p* = 0.0116), IL-2 (*p* = 0.0172; *p* = 0.0230), IL-6 (*p* = 0.0086; *p* = 0.0001), IL-10 (*p* = 0.0113; *p* = 0.0115), GM-CSF (*p* = 0.0362; *p* = 0.0359), TNF-α (NS; *p* = 0.0497), and CXCL1 (*p* < 0.0001; *p* < 0.0001) in the ABX group were higher than those in the control or ABX-FMT group. Twelve hours after CLP, the concentrations of IL-6 (*p* = 0.0392; *p* = 0.0388), IL-10 (*p* = 0.0452; *p* = 0.0428), and CXCL1 (*p* = 0.0489; *p* = 0.0482) in the ABX group exceeded those in the control or ABX-FMT group. Except for IL-2 concentrations in the ABX-FMT group being higher than those of the control group 12 hours after CLP (*p* = 0.0487), no significant difference in the concentrations of each cytokine at each time point was observed between the ABX-FMT and control groups (**Figure 6**).

Given that some standard deviations were relatively large, possibly due to small sample sizes, effect sizes were estimated using Cohen’s d and its 95% confidence interval for comparisons that reached statistical significance (*p* < 0.05). All such comparisons demonstrated large effect sizes (Cohen’s d > 0.8), indicating substantial differences between groups (**Supplementary Table S6**) (31).

### 3.7 Mortality caused by CLP-induced sepsis

**Figure 7:**
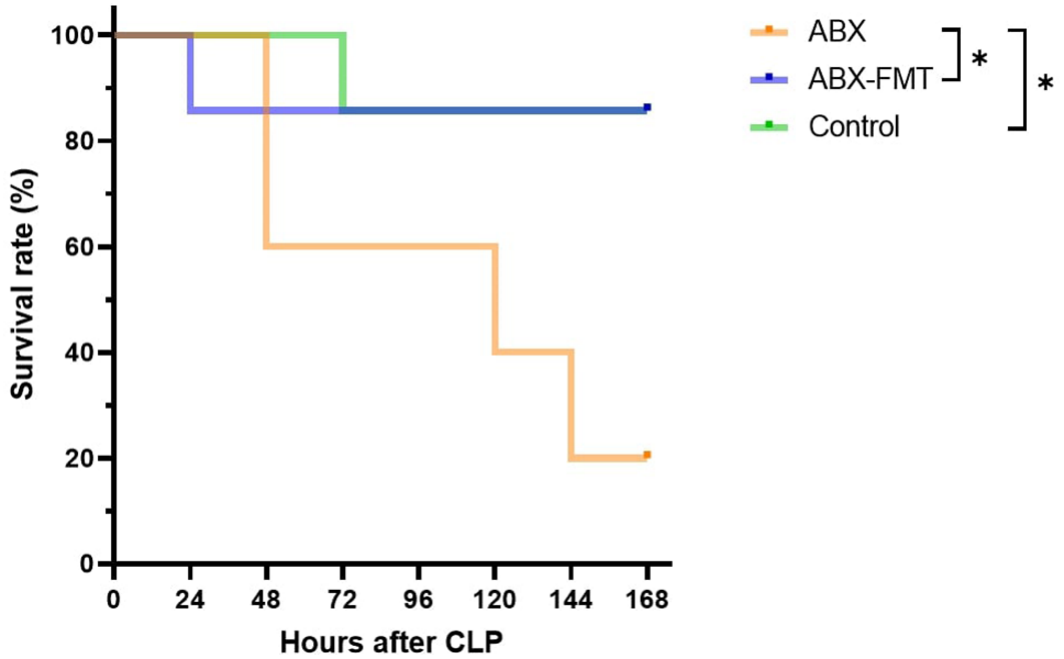
Kaplan-Meier survival curve. Sample sizes: *n* = 5 for the ABX group (two early deaths dropped out), and *n* = 7 for other groups. ABX, antibiotic treatment; FMT, fecal microbiota transplantation, *p < 0.05.

The survival rate after CLP was 20.0% (one out of five), 85.7% (six out of seven), and 85.7% (six out of seven) in the ABX, ABX-FMT, and control groups, respectively (ABX *vs.* control, *p* = 0.0267; ABX *vs.* ABX-FMT, *p* = 0.0461) (**Figure 7**). Two subjects in the ABX group that died within three hours post-CLP were excluded from the survival analysis.

### 3.8 Histopathological evaluation of the small intestines

**Figure 8:**
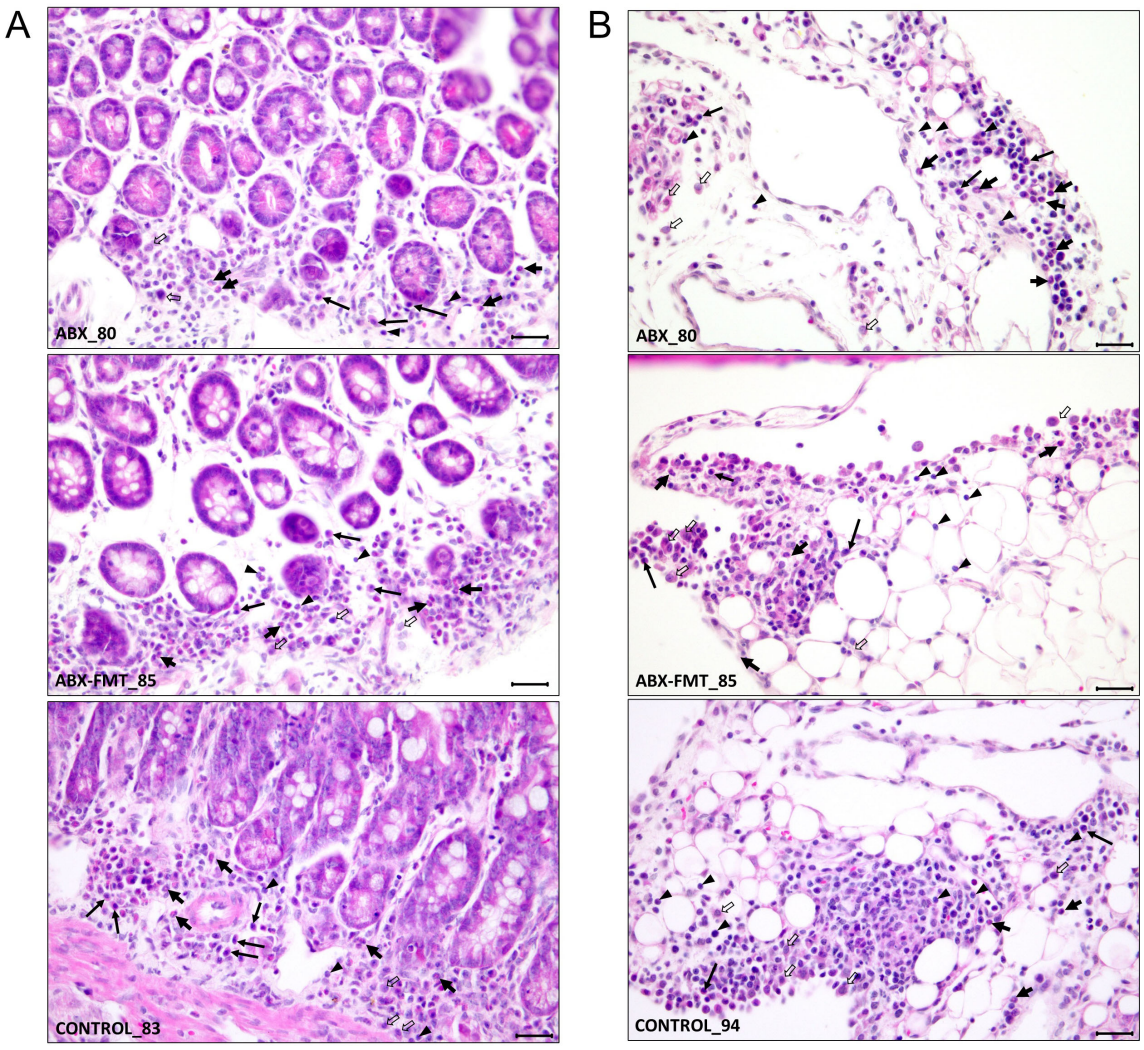
Representative histological features from each group showing the extent of infiltration of inflammatory cells. **(A)** Inflammatory cell infiltration in the lamina propria of the mucosa. **(B)** Inflammatory cell infiltration in the mesentery, indicating peritonitis. Annotations: neutrophils (thick short arrows), plasma cells (long arrows), macrophages (thick open arrows), and lymphocytes (arrowheads). Staining: H&E. Magnification: ×400; scale bars = 25 µm. ABX, antibiotic treatment; FMT, fecal microbiota transplantation.

The histological findings for each intestine are detailed in **Table 1**, summarized in **Table 2**, and illustrated with photos in **Figure 8**. In the ABX group, which had the lowest survival rate, infiltration of neutrophils, macrophages, lymphocytes, and plasma cells was confirmed in two microscopic examinations of the small intestine and peritoneum from a single mouse, with one mild and one moderate degree of inflammation. In the ABX-FMT group, neutrophils and macrophages were observed in nine examinations each, lymphocytes in six, and eosinophils and plasma cells in one examination each, across 12 microscopic examinations from six mice. In this group, the inflammation degrees were: three none, two minimal, six mild, and one moderate. In the control group, macrophages were observed in eight examinations, neutrophils and lymphocytes in six each, plasma cells in five, and eosinophils in one, across 12 microscopic examinations from six mice. Inflammation degrees in this group were: four none, four minimal, two mild, and two moderate. The Mann-Whitney test did not reveal significant differences in inflammation between the groups.

**Table 1:** Histopathological findings in the small intestines.

Group	ID	Histopathological findings
ABX	80	**Inflammatory cell infiltration**: Observed in the lamina propria and submucosa of the small intestine, moderate in extent. Infiltrating cells include neutrophils, lymphocytes, plasma cells, and macrophages. **Peritonitis**: Mild, characterized by infiltration of macrophages, plasma cells, lymphocytes, and neutrophils.
ABX-FMT	81	**Inflammatory cell infiltration**: Observed in the lamina propria and submucosa of the small intestine, mild in extent. Includes neutrophils, eosinophils, and macrophages. **Peritonitis**: Moderate severity, with infiltration of neutrophils, macrophages, and lymphocytes.
	85	**Inflammatory cell infiltration**: Mild infiltration observed in the lamina propria and submucosa of the small intestine, including neutrophils, macrophages, and lymphocytes. **Peritonitis**: Mild severity, characterized by the presence of macrophages, lymphocytes, and neutrophils.
	95	**Peritonitis**: Mild, with infiltration of macrophages, plasma cells, and neutrophils.
	511	No specific lesions observed.
	521	**Inflammatory cell infiltration**: Minimal infiltration in the lamina propria and submucosa of the small intestine, including neutrophils, macrophages, and lymphocytes. **Peritonitis**: Mild, with infiltration of macrophages, lymphocytes, and neutrophils.
	576	**Inflammatory cell infiltration**: Mild in the lamina propria and submucosa of the small intestine, with infiltration of neutrophils and macrophages. **Peritonitis**: Minimal, with infiltration of macrophages, lymphocytes, and neutrophils.
Control	82	**Inflammatory cell infiltration**: Minimal in the lamina propria and submucosa of the small intestine, with infiltration of neutrophils or eosinophils, lymphocytes, plasma cells, and macrophages. **Peritonitis**: Minimal, with infiltration of macrophages, plasma cells, and lymphocytes.
	83	**Inflammatory cell infiltration**: Moderate in the lamina propria and submucosa of the small intestine, with infiltration of neutrophils and macrophages.
	86	**Inflammatory cell infiltration**: Mild in the lamina propria and submucosa of the small intestine, with infiltration of neutrophils and macrophages. **Peritonitis**: Minimal, with infiltration of macrophages and neutrophils.
	89	**Peritonitis**: Minimal, with infiltration of plasma cells, lymphocytes, and macrophages.
	94	**Peritonitis**: Mild, with infiltration of macrophages, lymphocytes, plasma cells, and neutrophils.
	501	**Peritonitis:** Moderate, with infiltration of macrophages, lymphocytes, plasma cells, and neutrophils.

ABX, antibiotic treatment; FMT, fecal microbiota transplantation.

**Table 2:** Histological severity level for inflammatory lesions observed in the small intestine and peritoneum.

Group	ID	Extent of Inflammatory Cell Infiltration		Total number of samples corresponding to each severity level				
		Small intestine (lamina propria)	Peritoneum	NSL	Minimal	Mild	Moderate	Total
**ABX**	80	Moderate	Mild	0	0	1	1	2
**ABX-FMT**	81	Mild	Moderate	3	2	6	1	12
	85	Mild	Mild					
	95	NSL	Mild					
	511	NSL	NSL					
	521	Minimal	Mild					
	576	Mild	Minimal					
**Control**	82	Minimal	Minimal	4	4	2	2	12
	83	Moderate	NSL					
	86	Mild	Minimal					
	89	NSL	Minimal					
	94	NSL	Mild					
	501	NSL	Moderate					

ABX, antibiotic treatment; FMT, fecal microbiota transplantation; NSL, No specific lesion.

## 4 Discussion

This study investigated the effects of antibiotics on gut dysbiosis and the protective role of FMT against sepsis in juvenile mice. Characterized by their lower microbiome diversity and heightened sepsis susceptibility, juvenile mice were selected to align with clinical relevance in pediatric research.

In preliminary experiments using a standard diet for this study, a significant number of mice died within 48 hours post-CLP, leading to their exclusion from survival analyses. Both antibiotic groups exhibited lower food intake and weight gain rates particularly during antibiotic administration. Mice in any group with a body weight below 19–20 g on the CLP day showed higher mortality, despite no clear association between body weight and early unexpected deaths or final survival rates in mice exceeding 20 g. To address underweight-related concerns, several measures were implemented (1): enrolling mice with an initial weight of nine grams or more, a choice not particularly exclusive given that the average body weight of 3-week-old male mice is approximately 12 g and the average minus two standard deviations is about 7 g (32) (2), shortening the fasting time before oral gavage (3), adjusting anesthesia doses according to body weight, and (4) post-CLP warming. However, early unexplained deaths persisted in underweight mice. Introducing a high-fat diet in the main study aimed to mitigate these challenges, with no weight differences among groups on day 0, day 7, and day 14, as well as between surviving and dead mice (**Supplementary Table S7**).

The FMT method in this study was designed based on previously published studies. In humans, FMT is typically performed via tubes into the duodenum or jejunum (33), colonoscopy (34), or oral capsules (35), with 5 to 300 g of donated feces administered once or multiple times (36–38). The upper gastrointestinal approach is easier and less painful but should be avoided if there’s a high risk of regurgitation and aspiration pneumonia. The lower gastrointestinal approach requires colonoscopy, involving bowel preparation and risk of intestinal perforation.

In mice, previous studies administered 50 to 200 mg/kg of feces one to ten times at intervals of one to several days. Due to the difficulty of inserting a nasoduodenal tube in mice, the upper gastrointestinal approach used oral gavage, influenced by gastric acid and enzymes (39, 40). For lower gastrointestinal access, an enema method under mild anesthesia was employed (41, 42). In this study, to enhance FMT effectiveness, a higher dose of 500 mg/kg was administered seven times via oral gavage, successfully demonstrating bacterial engraftment.

Fecal microbiota at each time point displayed distinct characteristics based on the preceding interventions. This study identifies four types of fecal microbiota (1): healthy microbiota, dominated by the phyla Bacteroidetes and Firmicutes, persisted in the control group before CLP (2), antibiotic-induced dysbiota on day 7 in the ABX and ABX-FMT groups, primarily the class Bacilli (3), reconstituted dysbiota on day 14 in the ABX group, dominated by the phyla Verrucomicrobia and Proteobacteria, and (4) post-CLP microbiota on day 16 in the ABX-FMT and control groups, largely the phylum Bacteroidetes (**Figure 2**).

The microbiota of the control group before CLP was considered to be a healthy gut microbiota. To verify this, the influence of a high-fat diet must be taken into account. Although a high-fat diet over about two months can alter gut microbiota in mice, the two-week high-fat diet in this study was assumed not to cause significant changes. One study showed that obese mice had a low abundance of Bacteroidetes and a high abundance of Firmicutes, a pattern not seen in this study’s control group (43). While previous studies focused on adult mice and the composition of fecal microbiota in healthy mice varies across studies, Bacteroidetes and Firmicutes are consistently dominant phyla, similar to humans, aligning with the composition of the control group’s fecal microbiota in this study (44, 45). Moreover, the control group did not exhibit changes in fecal microbiota before CLP despite the high-fat diet, suggesting the diet did not significantly alter the microbiota. Although a high-fat diet was provided during the three-day acclimation period before collecting the first fecal samples on day 0, it is unlikely that the microbiota changed significantly only during the acclimation period and not over the subsequent 14 days.

This healthy gut microbiota was observed on day 0 of all mice and on day 7 and day 14 of the control group. Through FMT, it was reconstituted in the recipients’ guts, replicating the original composition, indicating successful FMT. Mice with this fecal microbiota immediately before CLP showed higher survival rates. The microbiota primarily consists of Bacteroidetes and *Bacteroides*, known for their anti-inflammatory effects, especially on inflammatory bowel disease (46, 47). *Bacteroides vulgatus*, the dominant species, is prevalent in human gut microbiota and is generally beneficial for colon health (48). Studies have shown its role in reducing inflammation and intestinal injury, though its impact on human immunity may vary depending on the strain (49–51).

*Bacteroides*, along with other gut microbiota members, produce microbe-associated molecular patterns (MAMPs) like flagellin, peptidoglycan, lipoteichoic acid, and LPS, recognized by pattern recognition receptors (PRRs). Precise regulation of PRR signaling is vital for maintaining healthy homeostasis (52). MAMPs, including peptidoglycan, can be released into the systemic circulation and prime the innate immune cells to respond efficiently to pathogens (53). Chemical analysis of *Bacteroides vulgatus* LPS revealed specific structural features crucial for its immunomodulatory effects, activating human macrophages *in vitro* and suggesting protection against pathogenic invasion (54).

As gut commensals, *Bacteroides* breaks down polysaccharides from dietary fibers and resistant starch into oligosaccharides or monosaccharides, which are then fermented by various intestinal microorganisms to produce short-chain fatty acids (SCFAs), including acetic acid, propionic acid, and butyric acid. These SCFAs serve as energy sources for intestinal cells, possess anti-inflammatory properties, regulate intestinal immunity, and maintain intestinal homeostasis (55).

*Parabacteroides goldsteinii*, a member of the family Porphyromonadaceae within the phylum Bacteroidetes, emerged as the second most dominant species in the healthy microbiota observed in this study. Recognized as a beneficial commensal bacterium for humans, it demonstrates protective effects against inflammatory conditions, including chronic obstructive pulmonary disease (56, 57).

Antibiotics generally induce unfavorable changes in gut microbiota by eliminating susceptible microorganisms, allowing resistant strains to overgrow and dominate. This destruction results in the loss of obligate anaerobes and a reduction in SCFAs, creating a conducive environment for potential pathogens (58, 59). This study observed a significant shift in Firmicutes, one of the two dominant phyla in the initial fecal microbiota, across all groups. Following antibiotic treatment, a notable imbalance occurred between the classes Clostridia and Bacilli, with Bacilli becoming the dominant class—comprising 90% of all classes—which led to a significant reduction in alpha diversity. The three genera primarily constituting the class Bacilli were *Lactococcus*, *Enterococcus*, and *Vagococcus*, with *Lactococcus fujiensis* being the most dominant species. It is presumed that antibiotic-resistant strains contributed to this drastic change in microbiota composition. Additionally, an increase in the abundance of unclassified species was observed, indicating further microbiota alterations.

Following antibiotic treatment cessation, the fecal microbiota of the ABX group underwent significant changes, including the replacement of once-dominant Bacilli with Clostridia, alongside notable increases in Verrucomicrobia and Proteobacteria. The high mortality in the ABX group may be linked to these microorganisms. However, determining the pathogenicity of Verrucomicrobia is complex. *Akkermansia muciniphila*, a mucin-degrading commensal offering anti-inflammatory benefits (60, 61), exhibited higher abundance in the higher CLP-mortality group mice, suggesting its potential role in inflammation by compromising intestinal mucosal barriers by reducing mucus protein (62).

Proteobacteria, housing numerous human pathogens such as *Brucella, Rickettsia, Bordetella, Neisseria, Escherichia, Pseudomonas, Shigella, Salmonella*, and *Yersinia*, appear to play a more substantial role in both extraintestinal and intestinal diseases compared to Verrucomicrobia (63). In a study on mice undergoing partial hepatectomy following a high-fat diet and antibiotic intake, high mortality, attributed to *Pseudomonas* sepsis, was observed, with a high cecal abundance of antibiotic-resistant Proteobacteria, including *Pseudomonas* (64). *Escherichia albertii*, a recently discovered species that potentially contributes to infectious diarrhea worldwide (65), appears to be the most pathogenic among the dominant species in this study. The secretome of *Escherichia albertii* comprises toxic components, such as toxins and proteins involved in adhesion and invasion, which elicit immune responses. These components can induce inflammation and immune activation, and, if the infection disseminates, may result in systemic effects (66, 67). Observations in a rat model of its translocation to mesenteric lymph nodes and liver suggest it as a likely causative microorganism for the lethal sepsis in most ABX-group mice (66).

After CLP, similar changes occurred in fecal microbiota of the ABX-FMT and control groups with higher survival rates. Of the two previously dominant phyla, Bacteroidetes and Firmicutes, only Bacteroidetes became excessively dominant, resulting in a substantial reduction of alpha diversity and making this microbiome resemble a pathobiome despite the higher survival of its hosts. It was not possible to compare this to fecal microbiota in the ABX group with lower survival rates since the feces of the ABX group were not able to be collected after CLP.

A study on a sepsis model of mice revealed that gut and peritoneal pathogens act as reservoirs for bloodstream infection, with fecal microbiota changes 24 to 48 hours post-CLP reflecting those in peritoneal lavage fluid and blood (68). The dominant presence of the genus *Bacteroides* (*Bacteroides vulgatus* or *Bacteroides stercorirosoris*) in day-16 fecal microbiota suggests their involvement in sepsis in the ABX-FMT and control groups. For the ABX group, based on the fecal microbiota immediately before CLP, it remains unclear whether the pathogens involved in sepsis belong to the class Clostridia, Verrucomicrobiae, or Gammaproteobacteria, as there was no information on the fecal microbiota after CLP. However, it can be assumed that the likelihood of the pathogens belonging to the phylum Bacteroidetes is very low, in contrast to other groups.

Immediate post-CLP changes in cytokine concentrations aligned with survival outcomes in each group. The ABX group exhibited a more rapid and pronounced increase in most pro-inflammatory cytokines compared to other groups, suggesting a stronger inflammatory responses (69). Anti-inflammatory cytokines, IL-4 and IL-10, play crucial roles in maintaining the balance of the inflammatory response and preventing tissue damage and organ failure due to excessive inflammation during sepsis (70, 71). While no significant difference in IL-4 concentrations was observed among the groups, the ABX-FMT group, which had a relatively high survival rate, demonstrated an increase in IL-4 and IL-10 levels over time post-CLP, suggesting that these cytokines may have contributed to modulating the inflammatory response. Conversely, the ABX group exhibited significantly higher IL-10 levels compared to the other groups, raising concerns that an excessive anti-inflammatory response may have led to immunosuppression.

Given the marked differences in microbiota between the groups with higher and lower survival rates, variations in the quantity and virulence of pathogenic microorganisms causing sepsis—stemming from differences in gut microbiota—may have contributed to the observed differences in cytokine responses. However, some significant comparisons in the cytokine analysis by Welch’s t-test had a lower bound of the 95% confidence interval for Cohen’s d below 0.5, indicating variability in the effect size estimates. This variability is likely due to the small sample sizes (seven per group, with two cases excluded in the ABX-FMT group pre-CLP), which can lead to wide confidence intervals. Consequently, the results of these comparisons should be interpreted with caution (**Supplementary Table S6**).

Histological changes in the small intestine and peritoneum were evaluated using simple H&E staining. Given that the time of organ removal varied for each deceased mouse and the pathological changes leading to death could be extensive, comparisons between groups would have been challenging if all deceased mice were included. Consequently, only mice that survived for seven days after CLP were analyzed, with only one mouse from the ABX group being included in the study. In the pathological findings, the ABX group exclusively exhibited mild to moderate inflammation, with no instances of none or minimal inflammation. In contrast, the ABX-FMT and control groups showed none or minimal inflammation in several cases, with mild to moderate inflammation also observed in some instances. Due to the limited sample size in the ABX group, statistical significance could not be determined.

This study has several limitations.

Firstly, some mice may have reached puberty by the conclusion of the study, which could potentially affect the results, although male mice are generally regarded as juveniles until approximately 6–8 weeks of age (72). In our experiment, antibiotic treatment, FMT, and CLP were all administered before the mice reached six weeks of age. However, since the mice were over six weeks old during the monitoring period after CLP, assessing gonadal development could have helped to ensure the exclusion of sexually mature mice from the analysis.

Secondly, the lack of post-CLP feces collection from the ABX group limits our understanding of the fecal microbiota in the group with higher mortality.

Thirdly, although we assumed that the high-fat diet would not introduce significant differences between groups or interfere with the sepsis process, the generalizability of this study’s results is debatable. In this study, each seven-day period of antibiotic intake or FMT was considered sufficient to create distinct conditions between groups. Therefore, further studies should enroll older juvenile mice to prevent underweight issues under standard diet conditions and employ a shortened treatment period to maintain their juvenile status until the study’s conclusion.

Fourthly, the relatively small sample size limited the statistical analysis. To improve the precision of effect size estimates and reduce uncertainty, future research should include larger sample sizes. Replicating these findings with more substantial sample sizes will help validate the observed effects and provide clearer insights into their practical significance.

Fifthly, due to the limitations of H&E staining, the observed infiltration of immune cells in the small intestine and peritoneum might not fully capture the complexities of the immune response in each group. H&E staining provides a general overview of tissue architecture and inflammation but has limitations in accurately identifying and differentiating specific immune cell types, such as neutrophils, macrophages, lymphocytes, and plasma cells, as well as in precisely identifying immune cell subtypes or assessing their functional states. Additional immunohistochemical staining or flow cytometry could offer more detailed insights into immune cell composition, activation states, and interactions within the tissue, thus complementing the findings obtained from H&E staining.

Finally, the absence of a sham-operated control group might have limited the study’s ability to obtain more convincing and reproducible results. Including such a group in future studies could strengthen the validity of the findings.

The safety of FMT in children has been partially demonstrated through clinical experience (73, 74). However, similar to adults, its indications are primarily limited to conditions like *Clostridium difficile* colitis and other intestinal diseases. Despite known differences in the intestinal microbiota between adults and children, FMT in children has mainly utilized feces donated by adults. There is a notable absence of research on the long-term effects of transplanting adult intestinal microorganisms on children’s immunity and metabolism. The efficacy of FMT from an age-matched donor, as demonstrated in this study, can serve as a foundation for actively pursuing research involving feces donated by children in clinical practice.

In addition, it should be noted that existing clinical or non-clinical studies involving FMT typically exclude the concurrent use of antibiotics for at least one day to ensure its effectiveness. However, when FMT is intended to restore the gut microbiota in patients with severe diseases requiring ongoing antibiotic treatment, preliminary research is necessary to assess the safety and efficacy of performing FMT simultaneously with antibiotic administration. Given that FMT must be conducted beyond the antibiotic administration period under these conditions, it is also crucial to consider the cost-effectiveness of such an approach. Additionally, for ease of application, a simple test—including a Gram stain or bacterial culture of feces—can be attempted to swiftly identify relevant patients (75).

In conclusion, this study highlights the successful replacement of antibiotic-induced fecal dysbiota with donor microbiota via FMT, providing a protective effect against sepsis. It uniquely confirms the efficacy of FMT in a juvenile mouse sepsis model, suggesting potential for clinical research strategies targeting critically ill children.

## Data Availability

The raw data supporting the conclusions of this article will be made available by the authors, without undue reservation.
