# Supplementary material for: Protective effect of gut microbiota restored by fecal microbiota transplantation in a sepsis model in juvenile mice
Source: Front Immunol. 2024 Oct 22;15:1451356. doi: 10.3389/fimmu.2024.1451356 (PMC11534669; doi:10.3389/fimmu.2024.1451356)
Supplement: Supplementary file 1 [file Table1.docx]

Supplementary Material

# Supplementary Data

## Methods in detail

### Cecal ligation and puncture (CLP) procedure

The mice were first anesthetized with 2.5% 2,2,2-tribromoethanol (250 mg/kg). Following anesthesia, the lower quadrants of the abdomen were shaved, and the surgical site was disinfected. A longitudinal midline incision was made using scissors, extending into the peritoneal cavity. After intramuscular, fascial, and peritoneal incisions, the cecum was located and exteriorized. The length of the cecum was measured at both ends of the ascending and descending portions. Subsequently, the cecum was ligated at 15 to 20% of its total length. To induce sepsis, one puncture was made in the cecum using a 20-gauge needle. Finally, the cecum was returned to its original position in the abdominal cavity, and the muscle tissue and skin were closed with sutures and medical clips, respectively.

### Post-CLP monitoring

After the surgery, body temperature was maintained using an infrared lamp. All mice were closely monitored for six hours following CLP. Immediately after the three-hour warming period, the body temperature was measured for mice that had not yet recovered from anesthesia. For mice with a body temperature of 32°C or lower, warming was continued, with body temperature measured at hourly intervals until six hours after CLP.

### Blood collection and preparation

The blood was collected from retroorbital venous plexus of anesthetized mice and stored at room temperature for 15–30 minutes before analysis. Subsequently, the blood was centrifuged at 17,000 × g for 20 minutes to obtain serum, which was then transferred to microtubes.

### Stool collection and storage

For stool collection, all mice were briefly manually restrained for defecation. One or two fecal lumps were collected using sterilized forceps. The collected feces were then stored in a freezer maintained at -20℃.

### Analysis of blood inflammatory biomarkers

The assays using the Bio-Plex 200 system were conducted following the manufacturer’s protocol (1). Plates were coated with anti-cytokine antibodies labeled with magnetic beads. Standards, serum samples, controls and blanks were pipetted into the corresponding wells and incubated for 30 minutes in the dark. Following the second washing step, biotinylated secondary antibodies were added. The plates were incubated in the dark for an additional 30 minutes and then subjected to the third washing step. Subsequently, the plates were incubated with the streptavidin-conjugated phycoerythrin for ten minutes in the dark. After a final washing step, the wells were resuspended in assay buffer. The plates were agitated for 30 seconds and measured using the Bio-Plex 200 system.

### Bacterial DNA extraction from feces

DNA extractions were conducted using the QIAamp DNA Stool Mini Kit (Qiagen, Germantown, MD, USA) following the manufacturer’s instructions. Collected feces were diluted with InhibitEX Buffer and homogenized through high-speed shaking using a Tissuelyser II (Qiagen) equipped with stainless steel beads (5 mm diameter, Ginormous Lab, Suwon, Korea). The sample matrix was pelleted by centrifugation and the DNA from the supernatant was purified using QIAamp Mini spin columns. Proteins were digested and degraded under denaturing conditions during incubation with proteinase K at 70℃. Buffer AL was then adjusted to ensure optimal DNA binding to the QIAamp membrane, and samples were loaded onto the QIAamp spin column. DNA was adsorbed onto the QIAamp silica membrane during a brief centrifugation step. DNA bound to the QIAamp membrane underwent two wash steps (Buffer AW1 and Buffer AW2). Purified and concentrated DNA was then eluted from a QIAamp Mini spin column using a low-salt buffer (Buffer ATE) [34, 35]. The yield of extracted DNA was determined using a NanoDrop 2000 spectrophotomether and a Qubit 3.0 fluorometer, both supplied by Thermo Fisher Scientific, Waltham, MA, USA (2).

### PCR amplification, Illumina MiSeq library preparation, and sequencing

The first PCR amplification involved initial denaturation at 94℃ for three minutes, followed by 25 cycles of 95℃ for 30 seconds, 55℃ for 30 seconds, and 72℃ for 30 seconds, with a final elongation step at 72℃ for five minutes. After the first PCR, duplicates were pooled and the amplified products were assessed using a 2100 Bioanalyzer Instrument (Agilent Technologies, Santa Clara, CA, USA). PCR cleanup was conducted using AMPure XP beads (Beckman Coulter Life Sciences, Indianapolis, IN, USA). The pooled products then served as the template for a second PCR (index PCR) with Nextera XT indexes (Illumina). The amplification conditions for the index PCR included an initial denaturation at 94℃ for three minutes, followed by eight cycles of 95℃ for 30 seconds, 55℃ for 30 seconds, and 72℃ for 30 seconds, with a final elongation step at 72℃ for five minutes. Samples with different indexes were quantified using the KAPA Library Quantification Complete Kit (Kapa Biosystems, Wilmington, MA, USA) and pooled in an equimolar ratio based on measured concentrations. The prepared library underwent further quality checks using a 2100 Bioanalyzer Instrument, and its concentration was measured with the Qubit dsDNA HS Assay Kit (Thermo Fisher Scientific) before proceeding to sequencing (2).

The library was diluted to a final concentration of 4 nM and 20% of PhiX DNA (Illumina) was added. Sequencing was carried out using the Miseq Reagent Kit V3 on a MiSeq 2000 instrument (Illumina), following the manufacturer’s instructions. The multiplexed sample underwent heat denaturation for two minutes at 96°C before loading onto the Illumina MiSeq chip. Following the 2 × 250 bp Illumina MiSeq paired-end sequencing run, the data were base-called, and reads with the same barcode were collected and assigned to their respective samples on the instrument, generating Illumina FASTQ files (2).

# Supplementary Tables

**Supplementary Table S1.** Taxa that showed significant differences in relative abundance over time in the ABX group

|  | *p*-value | |
| --- | --- | --- |
|  | Day 0 *vs*. day 7 | Day 7 *vs*. day 14 |
| *p__Bacteroidetes* | 0.0002 | NS |
| *c__Bacteroidia* | 0.0003 | NS |
| *g__Bacteroides* | 0.0031 | NS |
| *Bacteroides vulgatus* | 0.0056 | NS |
| *Bacteroides stercorirosoris* | 0.0254 | NS |
| *g__Parabacteroides* | 0.0024 | NS |
| *Parabacteroides goldsteinii* | 0.0024 | NS |
| *p__Firmicutes* | < 0.0001 | 0.0004 |
| *c__Clostridia* | 0.0072 | 0.0002 |
| *g__Clostridium* | NS | 0.0027 |
| *g__Alkaliphilus* | 0.0007 | NS |
| *g__Blautia* | 0.015 | 0.0016 |
| *c__Bacilli* | < 0.0001 | < 0.0001 |
| *g__Lactobacillus* | 0.0228 | NS |
| *g__Pediococcus* | 0.0335 | NS |
| *Pediococcus argentinicus* | 0.0334 | NS |
| *g__Lactococcus* | 0.0002 | 0.0002 |
| *Lactococcus fujiensis* | < 0.0001 | < 0.0001 |
| *g__Enterococcus* | 0.0014 | 0.0017 |
| *g__Vagococcus* | 0.0005 | 0.0006 |
| *Vagococcus penaei* | 0.0055 | 0.0055 |
| *p__Tenericutes* | 0.01 | NS |
| *p__Actinobacteria* | 0.0384 | 0.0253 |
| *c__Actinobacteria* | 0.0382 | 0.0255 |
| *p__Verrucomicrobia* | NS | < 0.0001 |
| *c__Verrucomicrobia* | NS | < 0.0001 |
| *g__Akkermansia* | NS | < 0.0001 |
| *Akkermansia muciniphila* | NS | < 0.0001 |
| *g__Rubritalea* | NS | < 0.0001 |
| *Escherichia albertii* | NS | 0.0413 |

ABX: antibiotic treatment, NS: not significant. Underlined *p*-values indicate a decrease in relative abundance, while *p*-values without an underline indicate an increase in relative abundance.

**Supplementary Table S2.** Taxa that showed significant differences in relative abundance over time in the ABX-FMT group

|  | *p*-value | | |
| --- | --- | --- | --- |
|  | Day 0 *vs*. day 7 | Day 7 *vs*. day 14 | Day 14 *vs*. day 16 |
| *p__Bacteroidetes* | <0.0001 | 0.0001 | 0.0015 |
| *c__Bacteroidia* | <0.0001 | 0.0001 | 0.0014 |
| *g__Bacteroides* | <0.0001 | 0.0002 | 0.0007 |
| *Bacteroides vulgatus* | 0.0012 | 0.0003 | NS |
| *Bacteroides stercorirosoris* | 0.0016 | 0.004 | 0.0007 |
| *g__Parabacteroides* | 0.0182 | 0.0162 | <0.0001 |
| *Parabacteroides goldsteinii* | 0.0022 | 0.0004 | NS |
| *p__Firmicutes* | <0.0001 | <0.0001 | 0.0015 |
| *c__Clostridia* | 0.0038 | 0.004 | 0.0014 |
| *g__Alkaliphilus* | 0.0046 | 0.0007 | 0.0007 |
| *Alkaliphilus crotonatoxidans* | 0.0049 | 0.0017 | 0.0016 |
| *g__Blautia* | 0.0057 | 0.0055 | 0.0051 |
| *c__Bacilli* | <0.0001 | <0.0001 | NS |
| *g__Lactobacillus* | 0.0032 | 0.0303 | 0.0293 |
| *Lactobacillus johnsonii* | 0.0127 | NS | NS |
| *g__Pediococcus* | 0.0006 | NS | NS |
| *g__Lactococcus* | <0.0001 | <0.0001 | 0.0031 |
| *Lactococcus fujiensis* | <0.0001 | <0.0001 | 0.0045 |
| *g__Enterococcus* | 0.0001 | 0.0001 | NS |
| *g__Vagococcus* | <0.0001 | <0.0001 | NS |
| *Vagococcus penaei* | <0.0001 | <0.0001 | 0.0055 |
| *p__Tenericutes* | 0.0007 | 0.0044 | NS |
| *p__Actinobacteria* | 0.0091 | 0.0168 | 0.0142 |
| *c__Actinobacteria* | 0.0085 | 0.0087 | 0.0075 |
| *p__Proteobacteria* | 0.0074 | 0.0071 | NS |
| *c__Gammaproteobacteria* | NS | 0.0067 | NS |

ABX: antibiotic treatment, FMT: fecal microbiota transplantation, NS: not significant. Underlined *p*-values indicate a decrease in relative abundance, while *p*-values without an underline indicate an increase in relative abundance.

**Supplementary Table S3.** Taxa that showed significant differences in relative abundance over time in the control group

|  | *p*-value | | |
| --- | --- | --- | --- |
|  | Day 0 *vs*. day 7 | Day 7 *vs*. day 14 | Day 14 *vs*. day 16 |
| *p__Bacteroidetes* | NS | NS | 0.0014 |
| *c__Bacteroidia* | NS | NS | 0.0014 |
| *g__Bacteroides* | NS | NS | 0.0008 |
| *Bacteroides vulgatus* | NS | NS | 0.0004 |
| *g__Parabacteroides* | NS | 0.0162 | <0.0001 |
| *Parabacteroides goldsteinii* | NS | 0.0162 | <0.0001 |
| *p__Firmicutes* | NS | NS | 0.002 |
| *c__Clostridia* | NS | NS | 0.0047 |
| *g__Clostridium* | NS | NS | 0.0203 |
| *g__Alkaliphilus* | NS | NS | 0.0007 |
| *Alkaliphilus crotonatoxidans* | NS | NS | 0.0016 |
| *g__Blautia* | NS | NS | 0.0053 |
| *c__Bacilli* | NS | NS | 0.0022 |
| *g__Lactobacillus* | NS | NS | 0.0033 |
| *Lactobacillus johnsonii* | NS | NS | 0.0078 |
| *g__Pediococcus* | NS | NS | 0.014 |
| *Pediococcus argentinicus* | NS | NS | 0.0141 |
| *p__Actinobacteria* | NS | NS | 0.0011 |
| *c__Actinobacteria* | NS | NS | 0.0013 |
| *p__Proteobacteria* | 0.0173 | NS | NS |

NS: not significant. Underlined *p*-values indicate a decrease in relative abundance, while *p*-values without an underline indicate an increase in relative abundance.

**Supplementary Table S4.** Taxa that showed significant differences in relative abundance between groups on Day 7

|  | *p*-value | |
| --- | --- | --- |
|  | ABX *vs*. control | ABX-FMT *vs*. control |
| *p__Bacteroidetes* | <0.0001 | <0.0001 |
| *c__Bacteroidia* | <0.0001 | <0.0001 |
| *g__Bacteroides* | <0.0001 | <0.0001 |
| *Bacteroides vulgatus* | <0.0001 | <0.0001 |
| *g__Parabacteroides* | <0.0001 | <0.0001 |
| *Parabacteroides goldsteinii* | <0.0001 | <0.0001 |
| *p__Firmicutes* | <0.0001 | <0.0001 |
| *c__Clostridia* | <0.0001 | <0.0001 |
| *g__Alkaliphilus* | <0.0001 | <0.0001 |
| *c__Bacilli* | <0.0001 | <0.0001 |
| *g__Lactobacillus* | <0.0001 | <0.0001 |
| *Lactobacillus johnsonii* | <0.0001 | <0.0001 |
| *g__Pediococcus* | <0.0001 | <0.0001 |
| *g__Lactococcus* | 0.0002 | <0.0001 |
| *Lactococcus fujiensis* | <0.0001 | <0.0001 |
| *g__Enterococcus* | 0.0014 | 0.0001 |
| *g__Vagococcus* | 0.0005 | <0.0001 |
| *Vagococcus penaei* | <0.0001 | 0.0054 |
| *p__Tenericutes* | 0.0002 | 0.0002 |
| *p__Actinobacteria* | 0.0005 | 0.0001 |
| *p__Verrucomicrobia* | NS | 0.0061 |
| *p__Proteobacteria* | NS | <0.0001 |

ABX: antibiotic treatment, FMT: fecal microbiota transplantation, NS: not significant. Underlined *p*-values indicate a higher relative abundance in the control group, while *p*-values without an underline indicate a lower relative abundance in the control group.

**Supplementary Table S5.** Taxa that showed significant differences in relative abundance between groups on Day 14

|  | *p*-value | |
| --- | --- | --- |
|  | ABX *vs*. control | ABX *vs*. ABX-FMT |
| *p__Bacteroidetes* | 0.0003 | 0.0001 |
| *c__Bacteroidia* | 0.0003 | 0.0001 |
| *g__Bacteroides* | 0.0008 | 0.0002 |
| *Bacteroides vulgatus* | 0.0008 | 0.0002 |
| *Bacteroides stercorirosoris* | 0.0041 | 0.0036 |
| *g__Parabacteroides* | <0.0001 | 0.0003 |
| *Parabacteroides goldsteinii* | 0.0003 | 0.0004 |
| *c__Clostridia* | 0.0281 | 0.0115 |
| *g__Clostridium* | 0.0037 | 0.0031 |
| *g__Alkaliphilus* | 0.0498 | 0.0119 |
| *g__Blautia* | 0.0085 | 0.0036 |
| *c__Bacilli* | 0.0089 | NS |
| *g__Lactobacillus* | 0.0031 | 0.028 |
| *Lactobacillus johnsonii* | 0.0078 | NS |
| *p__Actinobacteria* | 0.0341 | 0.039 |
| *p__Verrucomicrobia* | <0.0001 | <0.0001 |
| *c__Verrucomicrobia* | <0.0001 | <0.0001 |
| *g__Akkermansia* | <0.0001 | <0.0001 |
| *Akkermansia muciniphila* | <0.0001 | <0.0001 |
| *g__Rubritalea* | <0.0001 | <0.0001 |
| *g__Prosthecobacter* | 0.0295 | 0.0332 |
| *p__Proteobacteria* | 0.0236 | 0.0236 |
| *c__Gammaproteobacteria* | 0.0261 | 0.0391 |
| *Escherichia albertii* | 0.0402 | 0.0402 |

ABX: antibiotic treatment, FMT: fecal microbiota transplantation, NS: not significant. Underlined *p*-values indicate a lower relative abundance in the ABX group, while *p*-values without an underline indicate a higher relative abundance in the ABX group.

**Supplementary Table S6.** Comparison of significant mean differences, confidence intervals, *p*-values, and effect sizes (Cohen's d) for cytokine levels between and within groups across different times post-CLP using Welch's t-test

| Type of cytokine | Comparison | Difference of means | 95% CI for difference | *p*-value | Cohen’s d | 95% CI for Cohen's d |
| --- | --- | --- | --- | --- | --- | --- |
| IL-1β | 0h *vs.* 12h (ABX-FMT) | 30.34 | 19.28 to 41.40 | 0.0002 | 3.71 | 1.83 to 5.58 |
|  | 0h *vs.* 6h (ABX) | 3117.16 | 1010 to 5224 | 0.0111 | 1.93 | 0.67 to 3.20 |
|  | 6h *vs.* 12h (ABX) | 3050.61 | 943.4 to 5158 | 0.0122 | 1.89 | 0.63 to 3.15 |
|  | ABX *vs.* control at 6h | 3087.33 | 980.2 to 5194 | 0.0116 | 1.92 | 0.65 to 3.18 |
|  | ABX *vs.* ABX-FMT at 6h | 3085.69 | 978.5 to 5193 | 0.0116 | 1.92 | 0.65 to 3.18 |
| IL-2 | 0h *vs.* 12h (ABX-FMT) | 1.57 | 0.81 to 2.32 | 0.0022 | 2.73 | 1.14 to 4.30 |
|  | ABX *vs.* control at 6h | 4.56 | 1.12 to 8.00 | 0.0172 | 1.71 | 0.48 to 2.93 |
|  | ABX *vs.* ABX-FMT at 6h | 4.28 | 0.76 to 7.80 | 0.023 | 1.49 | 0.30 to 2.67 |
|  | ABX-FMT *vs.* control at 12h | 0.90 | 0.006 to 1.80 | 0.0487 | 1.17 | 0.04 to 2.31 |
| IL-4 | 0h *vs.* 12h (ABX-FMT) | 2.90 | 0.59 to 5.21 | 0.0192 | 1.62 | 0.30 to 2.93 |
|  | 6h *vs.* 12h (ABX-FMT) | 2.54 | 0.26 to 4.82 | 0.0318 | 1.30 | 0.15 to 2.45 |
| IL-6 | 0h *vs.* 12h (control) | 117.09 | 13.74 to 220.4 | 0.0323 | 1.48 | 0.30 to 2.66 |
|  | 0h *vs.* 12h (ABX-FMT) | 106.91 | 39.31 to 174.5 | 0.0083 | 2.07 | 0.65 to 3.48 |
|  | 0h *vs.* 6h (ABX-FMT) | 2115.06 | 39.86 to 4190 | 0.0469 | 1.33 | 0.07 to 2.60 |
|  | 0h *vs.* 6h (ABX) | 9487.00 | 9486 to 9488 | <0.0001 | 23238.3 | 14630.7 to 31845.9 |
|  | 6h *vs.* 12h (ABX) | 4706.04 | 364.9 to 9047 | 0.0379 | 1.42 | 0.25 to 2.59 |
|  | 0h *vs.* 12h (ABX) | 4780.96 | 439.8 to 9122 | 0.0358 | 1.44 | 0.26 to 2.62 |
|  | ABX *vs.* control at 6h | 6155.80 | 2230 to 10082 | 0.0086 | 2.05 | 0.76 to 3.34 |
|  | ABX *vs.* ABX-FMT at 6h | 7370.79 | 5296 to 9446 | 0.0001 | 4.65 | 2.63 to 6.66 |
|  | ABX *vs.* control at 12h | 4661.27 | 320.5 to 9002 | 0.0392 | 1.40 | 0.23 to 2.57 |
|  | ABX *vs.* ABX-FMT at 12h | 4673.48 | 332.8 to 9014 | 0.0388 | 1.41 | 0.24 to 2.58 |
| IL-10 | 0h *vs.* 12h (control) | 103.02 | 57.31 to 148.7 | 0.0011 | 2.84 | 1.36 to 4.33 |
|  | 0h *vs.* 6h (ABX-FMT) | 127.26 | 60.39 to 194.1 | 0.0034 | 2.48 | 0.96 to 4.00 |
|  | 0h *vs.* 12h (ABX-FMT) | 71.98 | 21.54 to 122.4 | 0.0126 | 1.85 | 0.49 to 3.22 |
|  | 0h *vs.* 6h (ABX) | 4568.44 | 1549 to 7588 | 0.0101 | 1.98 | 0.70 to 3.26 |
|  | 0h *vs.* 12h (ABX) | 2130.91 | 171.0 to 4091 | 0.0375 | 1.42 | 0.25 to 2.59 |
|  | ABX *vs.* ABX-FMT at 6h | 4434.37 | 1415 to 7454 | 0.0115 | 1.92 | 0.65 to 3.19 |
|  | ABX *vs.* control at 6h | 4448.23 | 1429 to 7468 | 0.0113 | 1.92 | 0.66 to 3.19 |
|  | ABX *vs.* ABX-FMT at 12h | 2052.13 | 92.25 to 4012 | 0.0428 | 1.37 | 0.21 to 2.53 |
|  | ABX *vs.* control at 12h | 2020.09 | 60.22 to 3980 | 0.0452 | 1.35 | 0.19 to 2.51 |
| IFN-γ | 0h *vs.* 12h (ABX-FMT) | 4.09 | 1.065 to 7.118 | 0.0135 | 1.69 | 0.36 to 3.02 |
|  | 0h *vs.* 6h (ABX) | 3.01 | 1.630 to 4.396 | 0.0017 | 2.84 | 1.36 to 4.33 |
|  | 6h *vs.* 12h (control) | 1.06 | 0.097 to 2.026 | 0.0337 | 1.29 | 0.14 to 2.44 |
| TNF-α | 0h *vs.* 12h (ABX-FMT) | 2.26 | 0.50 to 4.01 | 0.02 | 1.68 | 0.35 to 3.01 |
|  | 6h *vs.* 12h (ABX-FMT) | 1.88 | 0.11 to 3.64 | 0.0401 | 1.36 | 0.20 to 2.52 |
|  | 0h *vs.* 6h (ABX) | 55.72 | 0.48 to 111.0 | 0.0486 | 1.32 | 0.16 to 2.48 |
| GM-CSF | 0h *vs.* 12h (ABX-FMT) | 0.18 | 0.086 to 0.266 | 0.0021 | 2.47 | 0.95 to 3.98 |
|  | 0h *vs.* 6h (ABX) | 31.62 | 3.10 to 60.14 | 0.035 | 1.45 | 0.27 to 2.63 |
|  | 6h *vs.* 12h (ABX) | 29.32 | 0.79 to 57.85 | 0.0456 | 1.34 | 0.18 to 2.50 |
|  | ABX *vs.* ABX-FMT at 6h | 31.39 | 2.86 to 59.91 | 0.0359 | 1.44 | 0.26 to 2.61 |
|  | ABX *vs.* control at 6h | 31.31 | 2.7 to 59.83 | 0.0362 | 1.44 | 0.26 to 2.61 |
| CXCL1 | 0h *vs.* 12h (control) | 241.73 | 157.9 to 325.6 | 0.0004 | 3.76 | 2.02 to 5.51 |
|  | 0h *vs.* 6h (ABX-FMT) | 575.08 | 230.4 to 919.7 | 0.0065 | 2.18 | 0.74 to 3.62 |
|  | 0h *vs.* 12h (ABX-FMT) | 225.02 | 79.21 to 370.8 | 0.0092 | 2.02 | 0.62 to 3.62 |
|  | 0h *vs.* 6h (ABX) | 25620.06 | 22351 to 28889 | <0.0001 | 10.25 | 6.31 to 3.62 |
|  | 6h *vs.* 12h (ABX) | 20595.87 | 15392 to 25800 | <0.0001 | 4.67 | 2.65 to 6.70 |
|  | 0h *vs.* 12h (ABX) | 5024.19 | 278.5 to 9770 | 0.0412 | 1.38 | 0.22 to 2.55 |
|  | ABX *vs.* ABX-FMT at 6h | 25043.13 | 21774 to 28312 | <0.0001 | 9.97 | 6.13 to 13.80 |
|  | ABX *vs.* control at 6h | 24642.06 | 21287 to 27997 | <0.0001 | 8.99 | 5.50 to 12.48 |
|  | ABX *vs.* ABX-FMT at 12h | 4798.18 | 52.45 to 9544 | 0.0482 | 1.32 | 0.17 to 2.48 |
|  | ABX *vs.* control at 12h | 4777.57 | 31.89 to 9523 | 0.0489 | 1.32 | 0.16 to 2.47 |

ABX: antibiotic treatment, CLP: cecal ligation and puncture, CI: confidence interval, CXCL1: C-X-C motif chemokine ligand 1, FMT: fecal microbiota transplantation, GM-CSF: granulocyte macrophage colony-stimulating factor, IFN: interferon, IL: interleukin, TNF: tumor necrosis factor. Only comparisons with *p*-values less than 0.05 are included. Cells are shaded when the lower bound of the 95% confidence interval for Cohen's d is less than 0.5.

**Supplementary Table S7.** Body weight of mice in each group over time.

|  | Day 0 | | Day 7 | | Day 14 | |
| --- | --- | --- | --- | --- | --- | --- |
|  | Mean Wt (g) | SD | Mean Wt (g) | SD | Mean Wt (g) | SD |
| ABX (*n* = 7) | 15.7 | 0.57 | 20.4 | 1.88 | 23.0 | 1.34 |
| ABX-FMT (*n* = 7) | 16.2 | 0.78 | 19.9 | 1.83 | 21.8 | 1.58 |
| Control (*n* = 7) | 15.8 | 0.88 | 21.4 | 1.00 | 22.6 | 1.47 |
| Survivor (*n* = 13) | 15.9 | 0.87 |  |  | 22.2 | 1.56 |
| Non-survivor (*n* = 6) | 15.8 | 0.52 |  |  | 22.2 | 0.51 |

ABX: antibiotic treatment, FMT: fecal microbiota transplantation, SD: standard deviation, Wt: weight.

# References

1. Preuss JM, Burret U, Vettorazzi S. Multiplex Fluorescent Bead-Based Immunoassay for the Detection of Cytokines, Chemokines, and Growth Factors. *Methods Mol Biol*. (2021);2261:247-62. doi: 10.1007/978-1-0716-1186-9_15

2. Videnska P, Smerkova K, Zwinsova B, Popovici V, Micenkova L, Sedlar K, et al. Stool sampling and DNA isolation kits affect DNA quality and bacterial composition following 16S rRNA gene sequencing using MiSeq Illumina platform. *Sci Rep*. (2019);9:13837. doi: 10.1038/s41598-019-49520-3
